# Analysis of modulation factor to shorten the delivery time in helical tomotherapy

**DOI:** 10.1002/acm2.12075

**Published:** 2017-04-26

**Authors:** Hidetoshi Shimizu, Koji Sasaki, Hiroyuki Tachibana, Natsuo Tomita, Chiyoko Makita, Kuniyasu Nakashima, Kazushi Yokoi, Takashi Kubota, Manabu Yoshimoto, Tohru Iwata, Takeshi Kodaira

**Affiliations:** ^1^ Department of Radiation Oncology Aichi Cancer Center Hospital Nagoya Aichi Japan; ^2^ Department of radiation therapy education and research Graduate School of Radiological Technology Gunma Prefectural College of Health Sciences Maebashi Gunma Japan; ^3^ Department of Radiology Aichi Cancer Center Aichi Hospital Okazaki Aichi Japan

**Keywords:** delivery time, head and neck, helical tomotherapy, modulation factor, prostate

## Abstract

A low modulation factor (MF) maintaining a good dose distribution contributes to the shortening of the delivery time and efficiency of the treatment plan in helical tomotherapy. The purpose of this study was to reduce the delivery time using initial values and the upper limit values of MF. First, patients with head and neck cancer (293 cases) or prostate cancer (181 cases) treated between June 2011 and July 2015 were included in the analysis of MF values. The initial MF value (MF_initial_) was defined as the average *MF*_actual_ value, and the upper limit of the MF value (MF_UL_) was defined according the following equation: MF_UL_ = 2 × standard deviation of *MF*_actual_ value + the average *MF*_actual_

Next, a treatment plan was designed for patients with head and neck cancer (62 cases) and prostate cancer (13 cases) treated between December 2015 and June 2016. The average *MF*_actual_ value for the nasopharynx, oropharynx, hypopharynx, and prostate cases decreased from 2.1 to 1.9 (*p* = 0.0006), 1.9 to 1.6 (*p* < 0.0001), 2.0 to 1.7 (*p* < 0.0001), and 1.8 to 1.6 (*p* = 0.0004) by adapting the MF_initial_ and the MF_UL_ values, respectively. The average delivery time for the nasopharynx, oropharynx, hypopharynx, and prostate cases also decreased from 19.9 s cm^−1^ to 16.7 s cm^−1^ (*p* < 0.0001), 15.0 s cm^−1^ to 13.9 s cm^−1^ (*p* = 0.025), 15.1 s cm^−1^ to 13.8 s cm^−1^ (*p* = 0.015), and 23.6 s cm^−1^ to 16.9 s cm^−1^ (*p* = 0.008) respectively. The delivery time was shortened by the adaptation of MF_initial_ and MF_UL_ values with a reduction in the average *MF*_actual_ for head and neck cancer and prostate cancer cases.

## 1 Introduction

Helical tomotherapy (HT) is a delivery technique that modulates dose intensity using multileaf collimators (MLCs) of 64 leaves while synchronizing with the gantry rotation.^1^ The field width in the superior‐inferior direction of a patient is 5.0 cm at maximum; therefore, delivery time increases in cases with long target lengths. We have previously shown that delivery time decreases by adjusting parameters for dose optimization computing of the treatment plan.^2^ When a small value is set as the modulation factor (MF), that is one of the parameters, delivery time shortens; however, a small MF value results in poorer dose distribution.^2^, ^3^, ^4^ Therefore, it is necessary to set MF with a good balance of the delivery time and dose distribution. Because the proper setting of MF values varies across facilities and treatment sites,^5^ it is difficult to maintain a balance. A method has been proposed to search and adopt a lower setting of MF value while maintaining a good dose distribution by repeating the dose optimization computing with a lower setting of MF value for the completed treatment plan. However, some treatment planning system of tomotherapy (Accuray, Inc.) is not equipped with a graphics processing unit (GPU), and without GPU, the system takes more time for the dose optimization computing; thus, it is not effective to use this method for each patient. If a low MF value with good dose distribution maintenance is designed at the beginning of a treatment plan, the delivery time will be shortened and the treatment plan will be more efficient. We determined the optimal initial MF value by retrospective analysis of MF values used in the past. In addition, the upper limit of the MF value was used to avoid a larger setting of MF value than required. The purpose of this study was to reduce the delivery time by the initial value and upper limit value of MF.

## 2 Methods

### 2.A MF

MF is an index that expresses the complexity of the MLC motion. MF is defined by the following equation with the only beamlet (a radiation that passes an opened leaf) being used in the dose optimization computing:

(1) $$\mathrm{MF}\;\geq \;MF_{actual}\;=\;\frac{LOT_{\max}}{LOT_{\mathrm{average}}}$$

where *LOT*_max_ is the maximum leaf open time and *LOT*_average_ is the average leaf open time. The user sets a value (1.0–5.0) as MF in the design of a treatment plan. At the time of the dose optimization computing, MF lower than the preset value will be adopted as *MF*_actual_ because *LOT*_max_ is restricted; for example, if the setting MF is 2.0 and *LOT*_average_ of 200 ms is given, *LOT*_max_ can become 400 ms at maximum. If *LOT*_max_ is 390 ms, *MF*_actual_ becomes 1.95. Because the gantry rotation period of the tomotherapy is constant during beam delivery, shortening of the delivery time requires a shorter *LOT*_max_; therefore, a lower setting of MF is needed. If a leaf has an extremely long open time with a large setting MF, the open time of the leaf can be adopted as *LOT*_max_. In this case, even if the beamlet does not have a major impact on the dose distribution, the delivery time is idly longer because of the longer gantry rotation period.^6^

### 2.B Determination and adoption of initial MF value (MF_initial_) and upper limit of MF value (MF_UL_)

**Figure 1:**
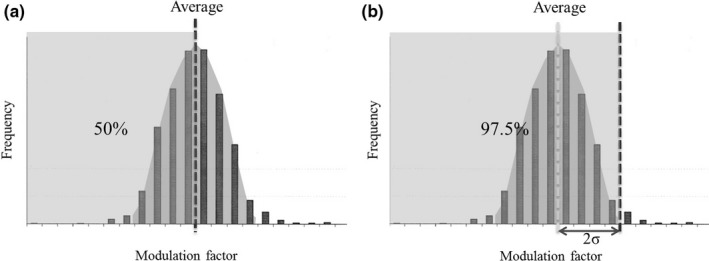
Definition of (a) initial modulation factor (MF) values (MF_initial_) and (b) upper limit of MF values (MF_UL_) for MF.

First, patients with head and neck cancer (293 cases) or prostate cancer (181 cases) treated using tomotherapy between June 2011 and July 2015 were analyzed. The primary sites of head and neck cancer were as follows: nasopharynx in 102 cases, oropharynx in 103 cases, and hypopharynx in 88 cases. The treatment plans were approved by two radiation oncologists, and it passed the dosimetry verification by a medical physicist and two radiation therapists. The delivery time and *MF*_actual_ were extracted from the treatment planning report. We hypothesized that the histogram of *MF*_actual_ would show normal distribution, so the average of *MF*_actual_ was defined as initial MF value (MF_initial_). The treatment plans of half of the overall cases could be statistically approved by the use of MF_initial_ (Fig. 1(a)). In addition, the value that added double of the standard deviation of *MF*_actual_ value to the average *MF*_actual_ was defined as the upper limit of the MF value (MF_UL_). Treatment plans of 97.5% of cases could be statistically approved by the use of MF_UL_ (Fig. 1(b)). It was hypothesized that 2.5% of the remaining MF values did not improve the dose distribution, whereas it extended the delivery time.

Second, treatment plans were designed for head and neck cancer (62 cases; 19 cases of nasopharynx, 17 cases of oropharynx, and 26 cases of hypopharynx) and prostate cancer (13 cases) treated using tomotherapy between December 2015 and June 2016. MF_initial_ was used for the treatment plan. If the dose distribution was not good, we increased the setting MF value up to MF_UL_ step by step in intervals of 0.1 or 0.2. As for the completed treatment plan, there was no problem in the clinic similar to the pre‐application of MF_initial_ and MF_UL_. In addition, it was confirmed that the plan quality (dosimetric parameter and dose distribution) in preapplication of MF_initial_ and MF_UL_ was equivalent to that in postapplication of MF_initial_ and MF_UL_.

The values of pitch and field width, which are the other treatment planning parameters, were 0.43 and 2.5 cm for all cases respectively. Chen et al. reported the reduction in a longitudinal dose ripple by using optimal pitch parameters;^7^ however, we used a conventional number (=0.86 /n, n; integer) that was proposed by Kissick et al.^8^ and it has been used routinely in many clinics. Furthermore, we confirmed that the longitudinal dose ripple effect was acceptable in each case. For the head and neck cancer cases, we conducted whole neck radiation, including the prophylactic lymph node region. For the prostate cancer cases, radiation was performed only for local sites (seminal vesicles and prostate).

### 2.C Data analysis

Because the delivery time was proportional to the amount of couch movement, which was approximately equal to the value that added the length of a planning target volume to the field width, the delivery time per amount of couch movement (s cm^−1^) was calculated; the distance of couch movement was extracted from the treatment planning report as well as the delivery time and *MF*_actual_. For *MF*_actual_ and delivery time in pre‐ and postapplication of MF_initial_ and MF_UL_, comparison of median values was calculated using Wilcoxon rank sum test. Statistical software R (Version 2.15.2) was used for all statistical analyses.^9^ *P* values < 0.05 were considered to be statistically significant.

## 3 Results

### 3.A Initial MF values (MF_initial_) and upper limit of MF values (MF_UL_)

Table 1 shows the average *MF*_actual,_ MF_initial_, and MF_UL_ for each treatment site. Because a preset of plural MF_initial_ values for the head and neck cancer cases might have induced an input mistake, we adopted 2.1, which was the largest average *MF*_actual_ in the nasopharynx, oropharynx, and hypopharynx cases, as the MF_initial_ in the head and neck cancer cases. MF_initial_ for the prostate cancer cases was 1.8, and it was lower than that for the head and neck cancer cases. Similarly, MF_UL_ for the head and neck cancer and prostate cancer cases were 2.6 and 2.2 respectively. For the preadaptation of MF_UL_, the percentage of *MF*_actual_ that was greater than the MF_UL_ in nasopharynx, oropharynx, hypopharynx, and prostate cases were 3.2%, 0.0%, 0.0%, and 2.8% respectively.

**Table 1:** Initial MF values (MF_initial_) and upper limit of MF values (MF_UL_) for each treatment site

	Average of actual MFs	MF_initial_	MF_UL_	Numbers above Mful
Nasopharynx	2.1	2.1	2.6	3/102
Oropharynx	1.9	2.1	2.6	0/103
Hypopharynx	2.0	2.1	2.6	0/88
Prostate	1.8	1.8	2.2	5/181

MF: modulation factor.

### 3.B Comparison of pre‐ and postadaptation of initial MF values (MF_initial_) and upper limit of MF values (MF_UL_)

**Figure 2:**
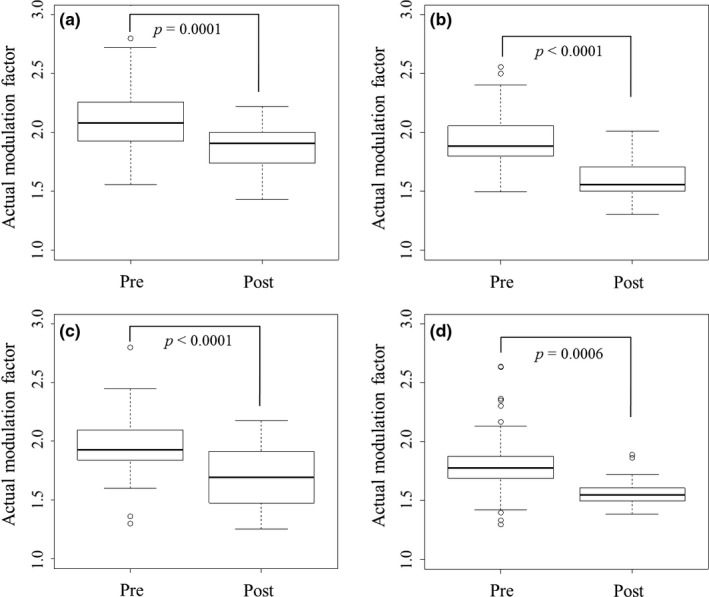
Actual modulation factors (MF) in pre‐ and postadaptation of initial MF values (MF_initial_) and upper limit of MF values (MF_UL_) (a) nasopharynx, (b) oropharynx, (c) hypopharynx, and (d) prostate.

Figure 2 shows *MF*_actual_ in pre‐ and post‐adaptation of MF_initial_ and MF_UL_. The average *MF*_actual_ for nasopharynx, oropharynx, hypopharynx, and prostate cancer cases decreased from 2.1 to 1.9 (*p* = 0.0006), 1.9 to 1.6 (*p* < 0.0001), 2.0 to 1.7 (*p* < 0.0001), and 1.8 to 1.6 (*p* = 0.0004) by the adaptation of MF_initial_ and MF_UL_ respectively. For the postadaptation of MF_UL_, the percentage of the *MF*_actual_ values less than the MF_initial_ values in nasopharynx, oropharynx, hypopharynx, and prostate cancer cases were 84.2%, 100.0%, 92.3%, and 84.6% respectively.

**Figure 3:**
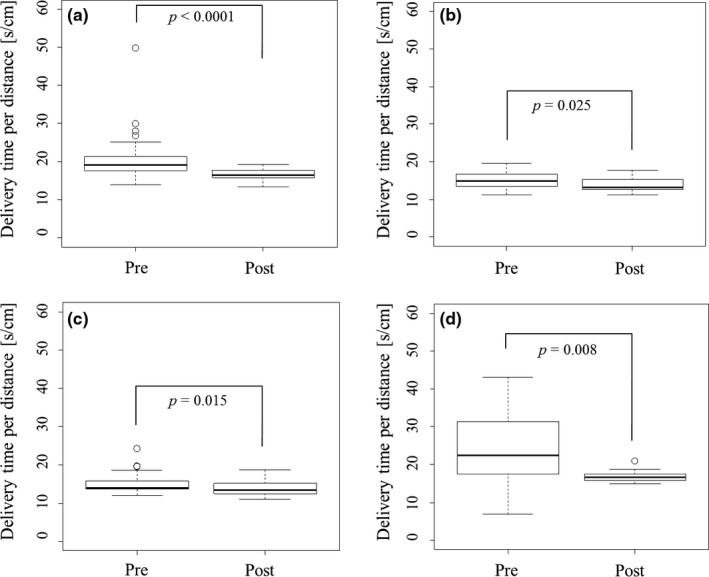
The delivery time per distance based on pre‐ and postadaptation of initial modulation factor (MF) values (MF_initial_) and upper limit of MF values (MF_UL_) (a) nasopharynx, (b) oropharynx, (c) hypopharynx, and (d) prostate.

Figure 3 shows the delivery time (s cm^−1^) in pre‐ and postadaptation of the MF_initial_ and MF_UL_ values. The average delivery time for nasopharynx, oropharynx, hypopharynx, and prostate cancer cases decreased from 19.9 s cm^−1^ to 16.7 s cm^−1^ (*p* < 0.0001), 15.0 s cm^−1^ to 13.9 s cm^−1^ (*p* = 0.025), 15.1 s cm^−1^ to 13.8 s cm^−1^ (*p* = 0.015), and 23.6 s cm = to 16.9 s cm^−1^ (*p* = 0.008) by the adaptation of the MF_initial_ and MF_UL_ values respectively.

## 4 Discussion

The average actual modulation factor and the average delivery time per distance (s cm^−1^) were significantly reduced by the introduction of an initial value and an upper limit value of the modulation factor, which was obtained by the analysis of the record of the treatment plan based on past values. A thermoplastic mask was fixed on the head, neck, and shoulders of patients with head and neck cancer. Patients with prostate cancer maintained full bladders to decrease bladder dose during the delivery time. The patients reported anxiety from the restriction of the mask or the leakage of urine, and shortening the delivery time reduced their anxiety. In addition, delivery time reduction also decreased the possibility that the patient would move during beam delivery. Hui et al. reported that the delivery time decreased to 75% by changing a preset of MF values from 2.5 to 2.0 for whole brain and whole craniospine.^3^ Skórska et al. found that the delivery time decreased with MF value reduction, although this finding was not statistically significant.^4^

Our method effectively reduced *MF*_actual_ values, and the average delivery time for nasopharynx, oropharynx, hypopharynx, and prostate cases decreased for 3.2 s cm^−1^, 1.1 s cm^−1^, 1.3 s cm^−1^, and 6.7 s cm^−1^ respectively. Table 2 shows the shortened delivery times; values were obtained by multiplying the shortened delivery time per distance (s cm^−1^) by the average couch movement distance for each treatment site. The delivery time for nasopharynx, oropharynx, hypopharynx, and the prostate cases was also shortened to 65.6 s, 24.0 s, 28.0 s, and 55.4 s respectively. The shortening effect of the delivery time for the oropharynx and hypopharynx cases was smaller than those of the nasopharynx and prostate cases, which likely resulted from defined MF_initial_ values from the average *MF*_actual_ values for the nasopharynx cases, although the average *MF*_actual_ values for nasopharynx cases is larger than that for the oropharynx and hypopharynx cases. If the MF_initial_ values from the average *MF*_actual_ values for oropharynx and hypopharynx cases are defined, a larger shortening effect on the delivery time is expected.

**Table 2:** Reduction in the delivery time

	Nasopharynx	Oropharynx	Hypopharynx	Prostate	Average
Couch movement distance [cm]	20.5	21.8	21.6	8.3	–
Decrease of delivery time per distance [s/cm]	3.2	1.1	1.3	6.7	–
Decrease of delivery time [s]	65.6	24.0	28.0	55.4	43.3

The proportion of *MF*_actual_ values less than the MF_initial_ values in postadaptation of the MF_initial_ values for nasopharynx, oropharynx, hypopharynx, and prostate cases were 84.2%, 100.0%, 92.3%, and 84.6% respectively. These results demonstrate that our method is effective in shortening the treatment plan because the frequency of MF value reset is low. The proportion of more than 50% estimated in Fig. 1(a) could be statistically obtained by the adoption of too large MF in past cases. Because MF used in our facility is a standard value used in Japan,^5^ it is likely that a good dose distribution can be obtained with shortened delivery time using our method in other facilities. Our method has versatility: if data accumulation of *MF*_actual_ values are available, our method can be easily performed in a facility; however, because the defined MF_initial_ and MF_UL_ values in this study were taken from a treatment planning protocol in our facility, the use of the values in other facilities must be thoroughly examined. In addition, the tomotherapy in our facility does not have a TomoEDGE^TM^ license, which uses dynamic jaw technology with dynamic adaptation of field width at cranial and caudal edges of a target.^10^ This technique can also shorten the delivery time by maintaining the quality of the dose distribution depending on the case.^10^, ^11^, ^12^ The use of the TomoEDGE™ is becoming more popular for head and neck cancer as well as for prostate cancer cases,^5^ and our method can further shorten the delivery time in combination with TomoEDGE^TM^.

## 5 Conclusions

Here, we defined an initial value and an upper limit value using a retrospective analysis of MF. The delivery time was shortened by the adaptation of these values with a reduction in the average *MF*_actual_ for head and neck cancer and prostate cancer cases.

## Conflict of interest

The authors declare no conflict of interest.
